# Mitochondrial-Dependent and Independent Functions of PINK1

**DOI:** 10.3389/fcell.2022.954536

**Published:** 2022-07-08

**Authors:** Xiusheng Chen, Qi Wang, Shihua Li, Xiao-Jiang Li, Weili Yang

**Affiliations:** Guangdong Key Laboratory of Non-human Primate Research, Guangdong-Hongkong-Macau Institute of CNS Regeneration, Jinan University, Guangzhou, China

**Keywords:** PINK1, mitophagy, mitochondria, Parkinson’s disease (PD), parkin (PARK2)

## Abstract

PINK1 has been characterized as a mitochondrial kinase that can target to damaged mitochondria to initiate mitophagy, a process to remove unhealthy mitochondria for protecting neuronal cells. Mutations of the human *PINK1* gene are also found to cause early onset Parkinson’s disease, a neurodegenerative disorder with the pathological feature of mitochondrial dysfunction. Despite compelling evidence from *in vitro* studies to support the role of PINK1 in regulation of mitochondrial function, there is still lack of strong *in vivo* evidence to validate PINK1-mediated mitophagy in the brain. In addition, growing evidence indicates that PINK1 also executes function independent of mitochondria. In this review, we discuss the mitochondrial dependent and independent functions of PINK1, aiming at elucidating how PINK1 functions differentially under different circumstances.

## Introduction

The PTEN-induced kinase 1 (PINK1) is a serine/threonine kinase whose function has been well characterized by biochemical studies and protein structural analysis (Kane et al., 2014; Kumar et al., 2017; Gan et al., 2022). A large amount of *in vitro* studies have shown that PINK1 works with Parkin, an E3 ubiquitin ligase, in coordination to target damaged mitochondria for removing unhealthy mitochondria by the lysosome, a process called mitophagy (Matsuda et al., 2010; Kane et al., 2014; Koyano et al., 2014; Ivankovic et al., 2016). In support of the role of PINK1/Parkin in mitochondria, mutations in the *PINK1* and *Parkin* genes are found to cause early onset of Parkinson’s disease that is also associated with mitochondrial dysfunction (Valente et al., 2004; Ishihara-Paul et al., 2008; McLelland et al., 2014; Ham et al., 2020). Identification of the involvement of PINK1/Parkin in mitophagy has expanded the roles of mitochondrial dysfunction and mitophagy in a variety of pathological conditions and diseases. As a result, extensive studies of the function of PINK1/Parkin related to mitochondrial function continue to provide a wealth of information about how PINK1/Parkin govern mitochondria homeostasis.

Despite the prevalent theory that PINK1 is a mitochondrial kinase and its mitochondrial-dependent function plays a critical role in the pathogenesis of PD, there have been unclear and important issues that need to be addressed. First, there is lack of strong *in vivo* evidence for PINK1/Parkin-mediated mitophagy in animal models. Second, non-mitochondrial dependent function of PINK1 has been reported, but whether this function is related to PD pathogenesis or other pathological conditions remains elusive. In this review, we will discuss the mitochondrial-dependent and non-dependent functions of PINK1, aiming at elucidating how PINK1 functions *in vivo* and how its dysfunction is involved in PD and other diseases.

### Parkinson’s Disease Is Associated With Mitochondrial Defects

Parkinson’s disease (PD) is the second most common neurodegenerative disorder (less prevalent than Alzheimer’s disease) and is characterized by age-dependent and progressive loss of neurons, especially dopamine neurons in the basal ganglia (Tolosa et al., 2006; Bloem et al., 2021). As a result of this selective neurodegeneration, PD is mainly manifested by motor dysfunction, accompanied by cognitive impairment and psychiatric abnormalities (Kalia and Lang, 2015). Pathologically, PD is featured by loss of dopamine neurons in the substantia nigra pars compacta region in the brain and accumulation of a-synuclein positive inclusions (Lewy bodies) (Bloem et al., 2021). The selective neurodegeneration in PD is thought to associate with mitochondrial dysfunction, which is supported by the discovery that mitochondrial toxins, such as 1-methyl-4-phenyl-1,2,3,6-tetrahydropyridine (MPTP), induce selective nigral degeneration in humans and animals (Fornai et al., 2005). Also, postmortem PD patient brain tissue display defects in mitochondrial bioenergetic capacity and function (Schapira et al., 1989; Schapira et al., 1990; Mallach et al., 2019).

Evidence to support the role of PINK1 in mitochondrial homeostasis also includes the association of genetic mutations of the *PINK1* gene with PD. Although most of PD patients start to develop symptoms when they reach the age over 50, early onset PD cases were also found in less than 10% of individuals with PD (Golbe, 1991). Most of these early onset cases are caused by genetic mutations, of which the autosomal recessive mutations in the *PINK1* gene were identified in early onset PD (Valente et al., 2004; Bonifati et al., 2005; Zhao et al., 2020). Biochemical analysis of PINK1 uncovers its function as a kinase that phosphorylates Parkin. Consistently, recessive mutations in the human Parkin gene were also found to cause early onset PD (Matsumine et al., 1997; Kitada et al., 1998). Furthermore, genetic studies of *Drosophila* harboring Pink1 mutations demonstrate that mitochondrial pathology caused by loss of Pink1 could be rescued by Parkin (Yang et al., 2006), establishing the theory that PINK1/Parkin act in the same pathway to protect mitochondria. Moreover, PINK1 deficiency in cellular models of PD has been reported to cause a loss of mitochondrial complex I reductive activity (Morais et al., 2014). Loss of functional mitochondrial complex I has been proved to be associated with dopaminergic cell death in PD (Surmeier et al., 2017; González-Rodríguez et al., 2021). However, complex I dysfunction is also implicated in sporadic PD, suggesting that complex I dysfunction can occur independent of PINK1 mutations.

### Mitochondrial Dependent Function of PINK1

The first *in vivo* evidence indicating that PINK1 is involved in regulating mitochondrial quality control came from genetic studies in *Drosophila*, which revealed that PINK1 null mutant flies showed apoptotic muscle degeneration, mitochondrial defects, and male sterility (Clark et al., 2006). Subsequently, a large body of biochemical studies have proved that PINK1 functions as a mitochondrial kinase. PINK1 is a 581 amino acid protein consisting of an N-terminal mitochondrial targeting motif that contains a transmembrane domain (110 amino acids long), a highly conserved kinase domain with three insertions in the N lobe, and a C-terminal autoregulatory sequence (Beilina et al., 2005; Cardona et al., 2011; Kumar et al., 2017). Mounting evidence from biochemical and *in vitro* studies indicates that PINK1 and Parkin work together in the same signaling pathway, as both proteins target damaged mitochondria to the lysosomes for clearance of the unhealthy mitochondria, a process called mitophagy (Pickrell and Youle, 2015; Ivankovic et al., 2016; Nguyen et al., 2016). The most compelling evidence to support the role of PINK1/Parkin in mitophagy is that PINK1 is targeted to mitochondria when cultured cells are under mitochondrial stress induced by the mitochondria-depolarizing agent such as CCCP (carbonyl cyanide m-chlorophenylhydrazine). Once PINK1 is localized on mitochondria, it phosphorylates Parkin and ubiquitin to recruit them to the damaged mitochondria, leading to the ubiquitination of mitochondrial proteins. Then the autophagic adaptor proteins such as p62/SQSTM1/sequestosome-1 were recruited to the damaged mitochondria and mediate the removal of damaged mitochondria by lysosomes (Nguyen et al., 2016). During this process, PINK1 acts as a key sensor of mitochondrial damage whereas Parkin amplifies this damage signal by facilitating the formation of ubiquitin chains, which recruit more Parkin to the damaged mitochondria (Harper et al., 2018). Further, analysis of the crystal structure of insect PINK1 bound to ubiquitin provides a structural base for the interactions of PINK1 with Parkin and ubiquitin (Kumar et al., 2017; Schubert et al., 2017; Gan et al., 2022). All these findings generate important insights into the function of PINK1 and strongly support the theory that PINK1 functions as a mitochondrial kinase and plays a pivotal role in mitophagy, an important intracellular process that is involved in a variety of cellular functions. Consistently, several lines of evidence indicate that PINK1 confers protection against mitochondria dependent apoptosis induced by both intrinsic stress and environmental insults (Gautier et al., 2008; Wang et al., 2011; Huang et al., 2017).

**FIGURE 1:**
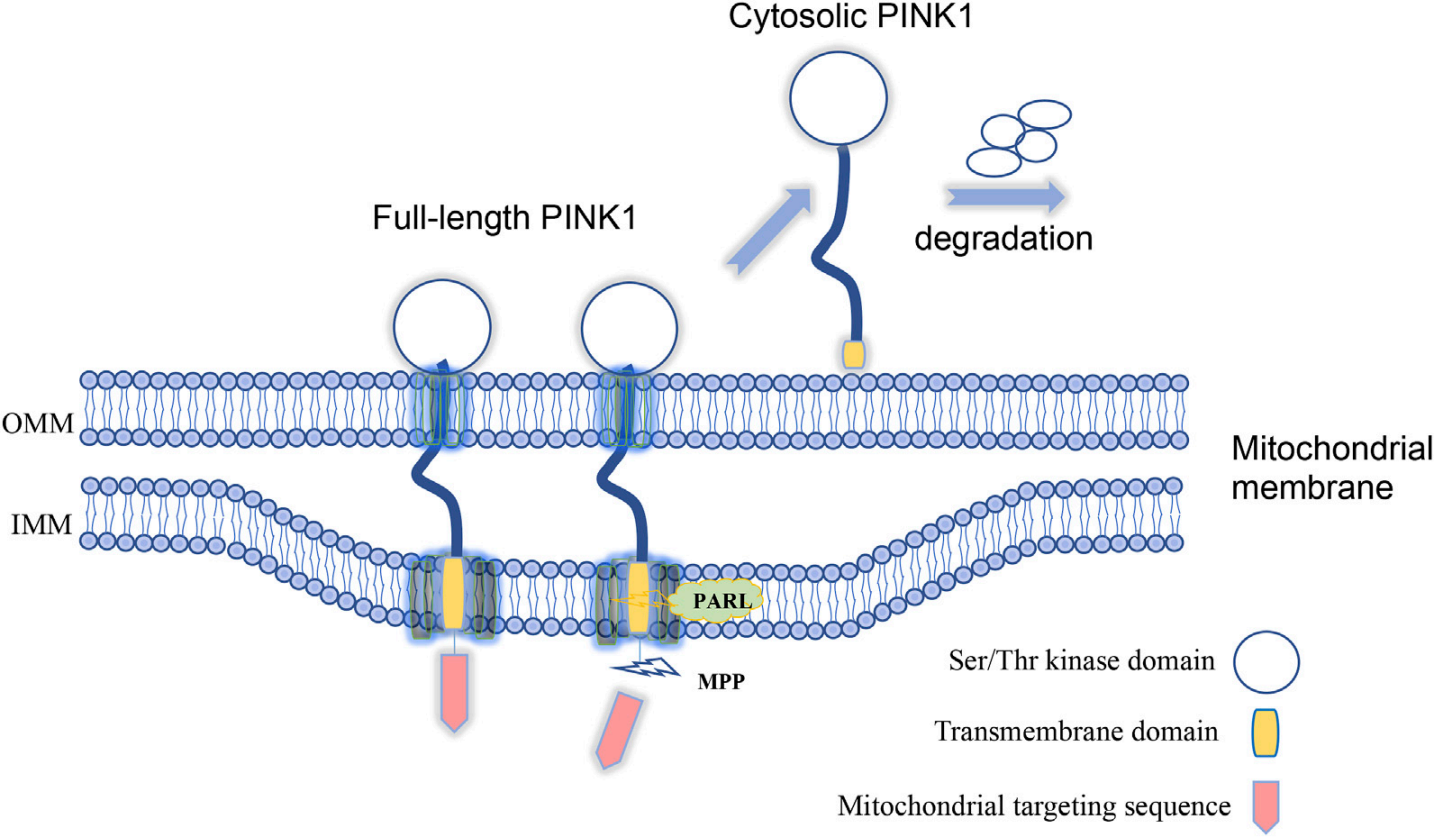
Full-length PINK1 is able to target to mitochondria and is cleaved by proteases to generate cytosolic form of PINK1 upon mitochondria damage. Lack of *in vivo* evidence for PINK1-mediated mitophagy.

Investigation of the function of PINK1 for mitophagy also yield important finding that endogenous PINK1 is synthesized constitutively in the cytosol as a full-length precursor (∼63–68 kDa). Upon import into mitochondria, PINK1 is proteolytically cleaved to produce its mature form (∼52–55 kDa) that is subsequently re-translocated to the cytosol, resulting in a rapid turnover and low steady state levels (Liu et al., 2017). Indeed, N-terminal region of PINK1, which is responsible for targeting PINK1 to the mitochondria, contains proteolytic sites that can be cleaved by MPP (mitochondrial processing peptidase) and PARL (presenilin-associated rhomboid-like protease) successively (Jin et al., 2010; Deas et al., 2011). These cleavages release PINK1 from mitochondria to produce a cytosolic form containing the kinase domain (Figure 1). However, when mitochondria are damaged, PINK1 is stabilized on the mitochondrial membrane, which can also be activated by kinetin that can amplify the catalytic activity of PINK1 (Hertz et al., 2013), to phosphorylate Parkin and ubiquitin to initiate mitophagy (Kane et al., 2014). All these findings convincingly demonstrate the mitochondrial-dependent function of PINK1 in cells. Since PINK1-mediated mitophagy is dependent on its targeting to mitochondria whereas cleavage of PINK1 via proteases can dissociate PINK1 from mitochondria and therefore inhibit PINK1’s function on mitophagy, suppressing proteases activity to cleave PINK1 is presumably able to enhance PINK1-dependent mitophagy process. In this regard, small-molecule inhibitors of the mitochondrial protease PARL that can cleave PINK1 could potentially enhance the activity of PINK1-depedendent mitophagy (Parsons et al., 2021).

### Lack of in vivo evidence for PINK1-mediated mitophagy

However, most of studies of PINK1 used cultured cells or *in vitro* systems to investigate mitochondrial-dependent function of full-length PINK1 in the field. Several groups have established PINK1 KO mouse models, aiming at identifying phenotypes associated with loss of PINK1 (Table 1). However, all these rodent KO models do not recapitulate the neurodegeneration seen in PD patient brains (Kitada et al., 2007; Akundi et al., 2011; Ge et al., 2020). Although rat PINK1 KO model was initially report to show DA neuronal degeneration (Dave et al., 2014), this phenotype is not severe as that in PD patient brain and was unable to be confirmed later by a different group (de Haas et al., 2019). Although PINK1/Parkin knockout mouse models are unable to show typical PD phenotypes, Parkin knockout could increase the vulnerability of dopaminergic neurons to exhaustive exercise via STING, a central regulator of the type I interferon response to cytosolic DNA (Sliter et al., 2018), suggesting that loss of Parkin alone is not sufficient to induce neurodegeneration in mice. To rigorously investigate the *in vivo* function of PINK1 for mitophagy, a knock-in mouse model, in which the codon encoding Parkin Ser65 was mutated to Ala65 to prevent its phosphorylation by PINK1, was established but this model still showed no clear neurodegeneration or nigrostriatal mitophagy impairment (McWilliams et al., 2018a).

**TABLE 1:** PINK1 knock out animal models

Model	Loss of dopaminergic neuron	Motor deficits	Mitophagy impairment	Reference
Mice	–	ND	ND	(Kitada et al., 2007)
Mice	–	ND	ND	(Akundi et al., 2011)
Rat	+	+	ND	(Dave et al., 2014)
Rat	–	+	ND	(de Haas et al., 2019)
Mice^a^	–	+	–	(McWilliams et al., 2018a)
Mice	ND	ND	–	(McWilliams et al., 2018b)
*Drosophila*	+	+	–	(Lee et al., 2018)
Rat	ND	ND	–	(Stauch et al., 2016)
Mice	ND	ND	–	(Yamada et al., 2019)
Pig	ND	ND	ND	(Zhou et al., 2015)
Pig	ND	–	ND	(Wang et al., 2016)
Monkey	+	+	–	(Yang et al., 2019a,b; Yang et al., 2022)
Monkey^b^ (adult)	+	+	ND	(Li et al., 2021)

ND: not detected

a This model was generated by *Parkin* Ser65Ala (S65A) knock-in to mimic PINK1 deficiency.

b This model was generated by co-editing *PINK1* and *DJ-1.*

Another strong evidence indicating that loss of PINK1 does not impact mitochondria homeostasis is the lack of influence of PINK1 on the basal mitophagy activity in *Drosophila* and mice (McWilliams et al., 2018b; Lee et al., 2018). In the PINK1 null fly and mice, *in vivo* mitophagy assay, which was performed using mito-QC or mt-Keima as a mitophagy reporter, did not show alteration as compared with wild type animals. Although the complex I subunit NDUFA10 was found to be phosphorylated by PINK1, transgenic overexpression of NDUFA10 can rescue *Drosophila* pink1 mutants independent of mitophagy (Pogson et al., 2014). Apart from these findings, mass spectrometry analysis of PINK1 knockout rodent did not show significant alterations in the expression levels of mitochondrial proteins (Stauch et al., 2016). As for mitochondrial function studies, inconsistent or mild alterations, at least not striking as *in vitro* findings, were found among the PINK1 KO animal models (Zhuang et al., 2016; Yamada et al., 2019). Also, PINK1/Parkin axis is not only the signaling pathway to regulate mitophagy, as growing evidence indicates the presence of PINK1/Parkin-independent mitophagy under *in vivo* or physiological conditions (Munson et al., 2021; Munson et al., 2022; Terešak et al., 2022). All these raise an important issue of whether PINK1 acts differentially *in vitro* and *in vivo* to mediate mitochondrial-dependent and independent functions.

Our recent studies using non-human primate model demonstrate for the first time that loss of PINK1 in the mammalian brain can cause neuronal loss (Table 1). We found that the deletion of the large *PINK1* DNA fragment by CRISPR/Cas9 can induce neuronal loss in the developing and adult monkey brains (Yang et al., 2019a; Yang et al., 2022). The homozygous deletion of a large region of the *PINK1* gene has not been found in humans, perhaps because such deletion is embryonic lethal in humans. However, CRISPR/Cas9-mediated deletion of the monkey *PINK1* gene can completely eliminate the expression of PINK1 to elicit severe neurodegeneration in the non-human primate brain (Yang et al., 2019a), which also suggests that *PINK1* point mutations found in patients with PD may partially impair PINK1 function to cause age-dependent neurodegeneration. One of the important findings from the non-human primate models is the striking neuronal loss without significant impact on mitochondria homeostasis (Yang et al., 2022). Also, the severe neuronal loss in the monkey brain is in clear contrast to the absence of neurodegeneration in mouse models that have completely deleted the *Pink1* gene, suggesting that PINK1’s function is species-dependent.

### Mitochondria Independent Function of PINK1

Although PINK1 is known to be cleaved to a truncated form by removing its N-terminal mitochondrial targeting domain, the functions of this cytosolic form of PINK1 have not been well characterized, and most of investigation focuses on the mitochondrial-dependent function of full-length PINK1 in the field. However, emerging evidence indicates that cytosolic PINK1 functions in many aspects to regulate cellular functions. In addition to PINK1-mediated phosphorylation of Parkin and ubiquitin (Eiyama and Okamoto, 2015), growing evidence indicates that the kinase activity of PINK1 spans to other substrates, including Drp1 (Han et al., 2020), TRAP1 (Pridgeon et al., 2007), Mfn2 (Chen et al., 2013), Miro (Wang et al., 2011), Bcl-xL (Arena et al., 2013), complex I subunit NdufA10 (Morais et al., 2014), and HtrA2 (Plun-Favreau et al., 2007). Phosphorylation of these signaling molecules appears to be independent of mitochondria but important for cell survival. In line with this idea, cytosolic PINK1 can mediate neuroprotection, since PINK1 lacking the mitochondrial targeting sequences, which can be produced by proteolytic process via N-end rule pathway, protects against MPTP-induced toxicity in mice (Haque et al., 2008). In addition, cytosolic PINK1 cannot promote mitophagy (Geisler et al., 2010; Narendra et al., 2010), suggesting that the pro-survival activity of PINK1 is not related to the mitophagy-inducing activity.

Investigation of PINK1-targeted monkey models also strongly supports the notion that PINK1 functions as a kinase *in vivo*, at least in the primate brains. Loss of PINK1 leads to marked decrease in phosphorylation of proteins that are important for neuronal survival (Yang et al., 2022). In support of this finding, the major form of PINK1 seen in the primate brain is the cytosolic form (55 kD) that lacks N-terminal region (Yang et al., 2022). This form of PINK1 contains the intact kinase domain and is presumably able to phosphorylate proteins in a mitochondrial independent manner. Earlier studies have revealed that cytosolic PINK1 promotes neuronal plasticity and differentiation, as PINK1-deficient cortical and midbrain neurons display defective dendritic morphology, and overexpression of truncated and cytosolic PINK1 could rescue this phenotype and also induce neuronal differentiation in SH-SH5Y neuronal cells (Dagda et al., 2014). The role of PINK1 in regulating neuronal differentiation is also supported by the finding from zebrafish and human organoid models that PINK1 deficiency impedes dopaminergic neuron neurogenesis (Brown et al., 2021).

PINK1’s function seems to be not restricted to the brain, as PINK1 is also upregulated in breast, colorectal, and endometrial cancer tissues, whereas PINK1 inhibition reduces cancer cell proliferation (Zhang et al., 2017). The cytosolic PINK1 has been implicated in signaling cascades critical to cell growth and survival, including the PI3-kinase (PI3K)/Akt, valosin-containing protein (VCP), and protein kinase A (PKA) pathways (Akundi et al., 2012; Soutar et al., 2018; Wang et al., 2018; Boonying et al., 2019). PINK1 deficiency in *Drosophila* is found to cause multiple growth defects independent of Parkin (Han et al., 2021). Along with the mitochondrial function of PINK1, the role of PINK1 in regulating cell cycle has also been reported, which is more likely to relate to tumor and cancer cells in which PINK1 expression is noticeably altered (O'Flanagan et al., 2015; Leites and Morais, 2018). PINK1 was initially identified as a downstream effector of phosphatase and tensin homolog (PTEN), a tumor suppressor that is frequently mutated in various types of human cancers (Unoki and Nakamura, 2001). Thus, more studies are required to investigate the mitochondrial-independent function of PINK1 and the relevance of this function to physiological and pathological scenarios.

### Questions That Need to Be Addressed

**FIGURE 2:**
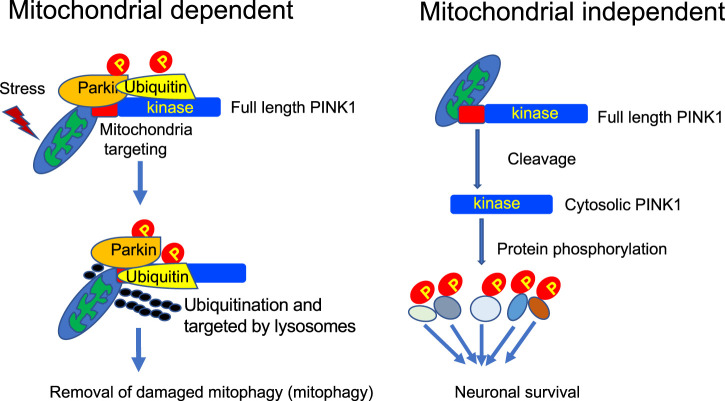
Mitochondrial-dependent and independent functions of PINK1. In the primate brain, PINK1 can phosphorylate a large number of proteins, including those for regulating mitochondrial function, to maintain neuronal survival.

The above description of mitochondrial-dependent and independent functions of PINK1 clearly indicates that PINK1 functions differently under different circumstances (Figure 2). It is difficult to reconcile the well-characterized mitophagy function of PINK1 and the absence of *in vivo* evidence for PINK1-mediated mitophagy in some animal models. It should be noted that most of *in vitro* studies of mitochondrial-dependent function of PINK1 involve overexpression of PINK1 or exogenously transfected PINK1 in combination with a mitochondrial depolarizing agent. It has been well recognized in the field that endogenous PINK1 is unstable and is very difficult to be detected in the rodent animals. This would explain that the intrinsically very low level of endogenous PINK1 is unable to recapitulate the *in vitro* function that is induced by overexpressed PINK1 with acute or extraordinary stress on mitochondria. On the other hand, endogenous PINK1 is more abundant in the primate brain and mainly functions as a kinase to phosphorylate neuronal proteins for maintaining the survival of primate neuronal cells. Thus, expression levels of PINK1 *in vivo* and *in vitro* are highly likely to account for different functions of PINK1. Also, as full-length PINK1 is more likely to act as a mitochondrial kinase whereas its cleaved products exist in the cytosol to function independent of mitochondria, the proteolytic processing of PINK1 determines the specific function of different PINK1 forms.

PINK1 seems to be a multifaceted protein acting at the crossroads of various pathways critical for cell survival, mitochondria quality control, and cell cycle regulation. The various functions of PINK1 raise many important issues that need to be well addressed and also pose challenges to our understanding of how these functions are regulated. First, why is there species-dependent expression of PINK1, for example, why is PINK1 undetectable in mice but abundant in the primates? The regulation of PINK1 expression is more likely mediated at translational and/or protein stability level, as *PINK1* mRNA is ubiquitously and abundantly expressed across different species (Blackinton et al., 2007). However, rigorous investigation of mechanisms underlying PINK1 protein expression and cleavage *in vivo* remains to be conducted. Lack of detectable expression of PINK1 in the rodent models makes it difficult to use small mammals to explore this important issue. Large animal models have been found to provide new insights into the pathogenesis of neurodegenerative diseases (Sun et al., 2022; Yin et al., 2022). However, the absent phenotypes of pig models that have deleted the *PINK1* gene (Zhou et al., 2015; Wang et al., 2016) suggest that endogenous PINK1 may also be expressed at a low level in the swine species. Thus, identification of the abundant expression of PINK1 kinase in the primate brains and the phenotypes due to PINK1 deficiency in the monkey model indicates that the non-human primates would be a suitable animal model for delineating the *in vivo* regulation of PINK1 expression and function.

Second, to what extent dose PINK1 mitochondrial-dependent function play a role *in vivo*, especially under pathological conditions? Mitophagy has been well documented and characterized for its protection against cell death. There is no doubt that PINK1/Parkin mediates mitophagy in cultured cells, but an important and outstanding question is whether this occurs *in vivo* when PINK1/Parkin are expressed at the endogenous level and under physiological conditions. Is full-length PINK1, which is able to localize to mitochondria, or cytosolic PINK1, which loses the ability to associate with mitochondria, more important for cell survival and function, and how do these different forms of PINK1 coordinate their functions *in vivo*?

Lastly, how important is PINK1’s function beyond mitochondria? Cytosolic PINK1 is evidently able to function as a kinase independent of mitochondria. Most kinases are key regulators of multiple cellular processes, and their functions and expressions are regulated by complex and sophisticated mechanisms whereas dysregulation of their expression can lead to diseases (Burke, 2018). If PINK1’s kinase function is critical for neuronal survival and differentiation, can improving its catalytic activity reduce neurodegeneration that is resulted from loss of PINK1 function? On the other hand, because PINK1 is upregulated in many tumor cells, can inhibition of its kinase activity suppress abnormal cell proliferation to treat tumorigenesis? Continued study of PINK1 beyond its mitochondrial functions may potentially shed light on novel therapeutic approaches.
